# Thrombolytic Therapy in High-Risk Pulmonary Embolism with Thrombocytopenia: Case Report and Literature Review

**DOI:** 10.3390/jcm15072569

**Published:** 2026-03-27

**Authors:** Marciana Ionela Boca, Alina-Ramona Cozlac, Caius Glad Streian, Simina Crisan, Mihai-Andrei Lazar, Mirela-Daniela Virtosu, Raluca Elisabeta Staicu, Dan Iliescu, Constantin-Tudor Luca

**Affiliations:** 1 Institute for Cardiovascular Diseases of Timisoara, “Victor Babeș” University of Medicine and Pharmacy of Timișoara, G. Adam Str. No. 13A, 300310 Timisoara, Romania; marciana.boca@umft.ro (M.I.B.); streian.caius@umft.ro (C.G.S.); simina.crisan@umft.ro (S.C.); lazar.mihai@umft.ro (M.-A.L.); daniela.cozma@umft.ro (M.-D.V.); raluca.staicu@umft.ro (R.E.S.); constantin.luca@umft.ro (C.-T.L.); 2 Doctoral School Medicine-Pharmacy, “Victor Babeș” University of Medicine and Pharmacy of Timișoara, Eftimie Murgu Square No. 2, 300041 Timișoara, Romania; 3 Advanced Research Center of the Institute for Cardiovascular Diseases, “Victor Babeș” University of Medicine and Pharmacy of Timișoara, Eftimie Murgu Square No. 2, 300041 Timișoara, Romania; 4 Department VI Cardiology-Cardiovascular Surgery, “Victor Babeș” University of Medicine and Pharmacy of Timișoara, Eftimie Murgu Square No. 2, 300041 Timișoara, Romania; 5 Department of Surgery I—Clinic of Surgical Semiotics & Thoracic Surgery, Center for Hepato-Biliary and Pancreatic Surgery, “Victor Babeș” University of Medicine and Pharmacy of Timișoara, Eftimie Murgu Square, No. 2, 300041 Timisoara, Romania; dan.iliescu@umft.ro

**Keywords:** pulmonary embolism, thrombocytopenia, thrombolysis, alteplase, heparin-induced thrombocytopenia, high-risk pulmonary embolism

## Abstract

**Background/Objectives**: Pulmonary embolism (PE) is a major cause of cardiovascular mortality, particularly in high-risk cases complicated by hemodynamic instability. Systemic thrombolysis is the recommended treatment in such settings; however, the coexistence of thrombocytopenia represents a major therapeutic challenge due to concerns regarding bleeding risk. Evidence guiding thrombolytic therapy in thrombocytopenic patients with PE is limited. This study aimed to present a representative case and review the available literature addressing thrombolysis in PE complicated by thrombocytopenia. **Methods**: A qualitative review of published case reports was conducted using the PubMed and Scopus databases, and articles describing adult patients with objectively confirmed PE, documented thrombocytopenia, and treatment with thrombolytic therapy were included. Eight case reports met the inclusion criteria, and the clinical characteristics, severity markers, platelet dynamics, treatment strategies and outcomes were analyzed and compared with the reported case. **Results**: Most of the reported patients presented with high-risk pulmonary embolism, defined by hemodynamic instability, including shock or cardiac arrest. Thrombolysis was frequently administered despite platelet counts below conventional thresholds. Platelet levels at the time of thrombolysis varied widely, including cases of severe thrombocytopenia. Clinical and hemodynamic improvement was observed in most of the cases, while major bleeding complications were infrequent. The reported case demonstrated successful systemic thrombolysis with rt-PA in a postpartum patient with suspected heparin-induced thrombocytopenia and high-risk PE, without hemorrhagic events. **Conclusions**: Available evidence suggests that thrombolytic therapy may be a viable life-saving option in carefully selected thrombocytopenic patients with high-risk pulmonary embolism. Therapeutic decisions should prioritize clinical severity and hemodynamic status over platelet count alone, emphasizing individualized, multidisciplinary risk–benefit assessment.

## 1. Introduction

Pulmonary embolism (PE) represents a major cause of cardiovascular mortality and morbidity worldwide, with clinical outcomes largely determined by risk stratification [1,2]. Venous thromboembolism (VTE), which encompasses deep venous thrombosis and PE, is recognized as the third most frequent acute cardiovascular disorder [3]. Risk assessment of PE is primarily based on the severity of hemodynamic compromise and the presence of right ventricular dysfunction [4]. Patients with high-risk PE, characterized by hemodynamic instability or shock, are associated with a markedly increased risk of early mortality and therefore require prompt reperfusion therapy [5]. In this setting, systemic thrombolysis remains the cornerstone of treatment and is strongly recommended by current international guidelines [1]. Patients with intermediate-risk PE require close clinical and hemodynamic surveillance to allow early identification of clinical deterioration and timely initiation of rescue reperfusion therapy when indicated [6].

Despite its established efficacy, systemic thrombolytic therapy is associated with a substantial risk of bleeding and is therefore subject to well-recognized absolute and relative contraindications, including recent major surgery, active bleeding, intracranial pathology, and severe thrombocytopenia [7,8]. These limitations complicate clinical decision-making in patients with coexisting conditions that predispose to hemorrhage [9,10].

Thrombocytopenia represents a major clinical limitation in the context of thrombolytic therapy, as platelet counts below defined thresholds are considered a contraindication in several clinical settings [11]. In particular, a platelet count < 100,000/mm^3^ is listed as a contraindication to thrombolytic therapy for PE [9,12]. However, the evidence underlying this recommendation is limited, as hemorrhagic complications associated with thrombolysis in thrombocytopenic patients have not been systematically evaluated in prospective studies or randomized clinical trials [13]. Available data are restricted to a very small number of reported cases, limiting definitive conclusions regarding the safety of thrombolytic therapy in this population and underscoring that current recommendations are largely derived from expert consensus rather than high-quality clinical evidence [9]. In the absence of prospective studies, detailed case reports and qualitative syntheses of published experience remain essential to guide management in this rare and high-risk clinical setting. Although evidence remains limited, several systematic reviews have explored the complex therapeutic balance between thrombotic risk and bleeding risk in patients with pulmonary embolism and thrombocytopenia [14].

We present a representative case of high-risk pulmonary embolism complicated by thrombocytopenia and provide a narrative review of published cases to highlight clinical severity patterns, platelet dynamics, therapeutic strategies, and outcomes.

## 2. Materials and Methods

This article presents a clinical case report accompanied by a qualitative review of published case reports describing thrombolytic therapy in adult patients with pulmonary embolism and thrombocytopenia. The review was not designed as a formal systematic review or meta-analysis. The literature review was performed using the PubMed and Scopus databases, with the study selection being guided by predefined inclusion and exclusion criteria with the aim of identifying published case reports describing the use of thrombolytic therapy in patients diagnosed with PE who also presented thrombocytopenia.

To maximize search accuracy, an advanced strategy was applied using the Boolean operator “AND”, combining the terms (“pulmonary embolism” AND thrombocytopenia AND (thrombolysis OR thrombolytic OR alteplase OR “rt-PA”)). Additional keyword combinations and medical subject headings (MeSH) were also used to increase search specificity and ensure comprehensive retrieval of relevant articles.

In total, 110 records were initially identified. Following removal of duplicates and exclusion of articles without available full text, not written in English, being published before 2009, or not defined as case reports, 8 studies met the inclusion criteria and were selected for qualitative synthesis.

To ensure clinical relevance and comparability with the reported case, we identified case reports published between 2009 and 2025 with full-text access, being written in English. The inclusion criteria required that articles report adult patients with objectively confirmed PE, documented thrombocytopenia at presentation or prior to thrombolytic therapy, clearly described treatment strategies, and explicit clinical outcomes. Only studies defined as case reports and published within the predefined study period were considered eligible. Exclusion criteria comprised articles published outside the specified time frame, studies other than case reports, reports involving non-human participants, and studies lacking sufficient clinical or therapeutic detail. Given the nature of this study as a qualitative review of case reports, no formal quality assessment tools were applied; however, all included reports were evaluated for completeness of clinical data, diagnostic confirmation, and treatment description.

All identified references were imported into the Zotero reference management software, and duplicate entries were removed. Data extraction and manuscript preparation were performed using Microsoft 365 (Office) software, Microsoft Corporation, Redmond, WA, USA.

## 3. Results

The case report illustrates a 34-year-old woman, a former smoker, presented with a complex thromboembolic clinical course in the postpartum period. Following her second childbirth, she developed acute massive left iliofemoral thrombophlebitis, for which she was admitted to a local hospital, and anticoagulant therapy with enoxaparin was initiated. The platelet count prior to initiation of anticoagulation was 124,000/µL. Four days later, despite ongoing anticoagulation, the patient developed an acute PE. Computed tomography pulmonary angiography (CTPA) was performed, revealing near-complete obstructive endoluminal thrombi within the right pulmonary artery, involving the right lower lobar branch, as well as thrombotic occlusion of the left terminal pulmonary artery and the left lower lobar branch, confirming the diagnosis of massive bilateral PE. In response, anticoagulant therapy was escalated to continuous intravenous unfractionated heparin. Subsequently, the patient’s clinical condition deteriorated, with the onset of hemodynamic instability and arterial hypotension, requiring positive inotropic support with norepinephrine. She was therefore transferred to our clinic for further management.

At admission, the patient presented in severely altered general condition. Cardiovascular examination revealed regular rhythmic heart sounds and a grade II/VI systolic murmur best heard at the tricuspid area. Pulmonary auscultation revealed normal breath sounds, accompanied by tachypnea. Peripheral oxygen saturation with room air was 89%. Physical examination of the left lower limb revealed marked edema with local inflammatory signs, consistent with ongoing deep venous thrombosis.

**Figure 1:**
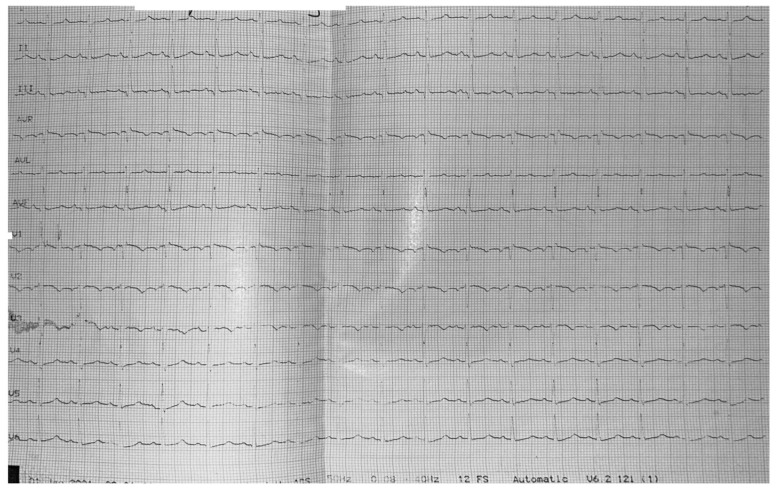
Twelve-lead electrocardiogram obtained at admission, showing S1Q3T3 pattern.

Resting electrocardiography (ECG) revealed sinus rhythm with a heart rate of 105 beats per minute, right axis deviation, and a classic S1Q3T3 pattern, suggestive of acute right ventricular strain, as shown in Figure 1.

Laboratory investigations at admission demonstrated moderate normocytic, normochromic anemia (hemoglobin 8.7 g/dL), moderate thrombocytopenia (platelet count 53,000/µL), and evidence of an inflammatory response, with an erythrocyte sedimentation rate of 25 mm/h. A mild leukocytosis was present (white blood cell count 11,340/µL) with associated neutrophilia. Cardiac biomarkers showed a mild elevation of high-sensitivity troponin I (31.3 ng/mL), while D-dimer levels were markedly increased (7650 ng/mL), supporting the diagnosis of acute PE.

**Figure 2:**
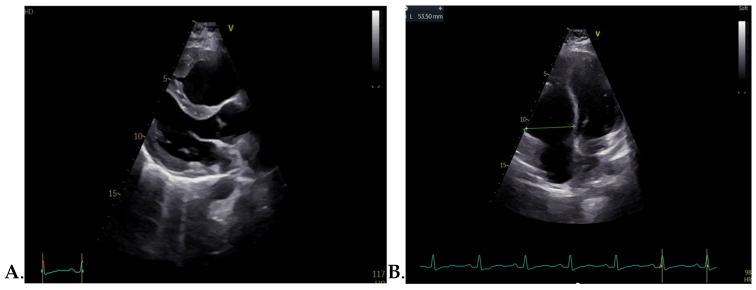
Transthoracic echocardiography at admission demonstrating marked right ventricle dilatation (**A**) Parasternal shorts axis view (**B**) Apical four chamber view.

**Figure 3:**
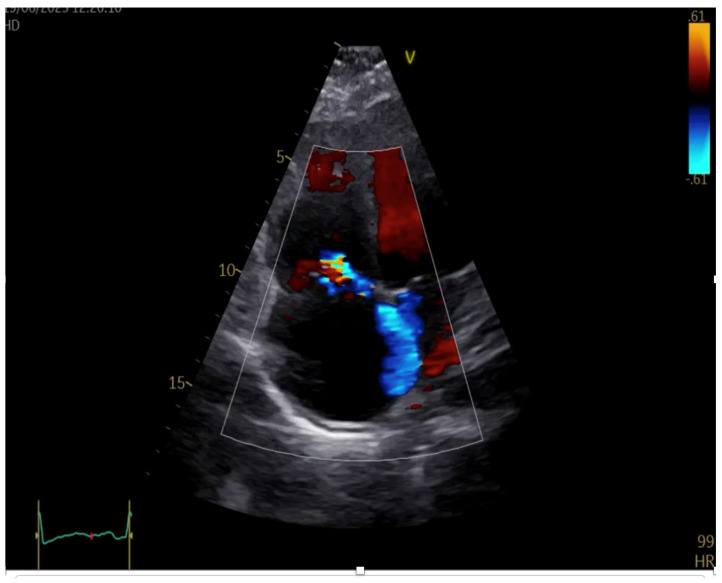
Transthoracic echocardiography at admission, apical four chamber view, showing severe tricuspid regurgitation.

Transthoracic echocardiography performed at admission revealed marked dilatation of the right heart chambers, exceeding left-sided cavity dimensions, accompanied by reduced longitudinal systolic function of the right ventricle (RV) and an estimated pulmonary artery pressure (PAP) of 60 mmHg, as shown in Figure 2. Severe functional tricuspid regurgitation was identified, as shown in Figure 3. In contrast, the left ventricle exhibited normal dimensions and preserved systolic function, with only mild mitral regurgitation.

Risk stratification was performed using validated prognostic scores. The Pulmonary Embolism Severity Index (PESI) score was 124, and the simplified PESI (sPESI) score was 3, classifying the patient as high risk, with an increased likelihood of early mortality and adverse clinical outcomes.

Following comprehensive risk stratification, the clinical assessment concluded that the risk of sudden cardiac death outweighed the potential risk of hemorrhagic complications. Consequently, a decision was made to initiate systemic thrombolytic therapy with recombinant tissue plasminogen activator (rt-PA). Systemic thrombolysis with alteplase was initiated with a 10 mg intravenous bolus, followed by infusion of 40 mg at 40 mL/h and subsequently 50 mg at 50 mL/h, resulting in a total dose of 100 mg. This was subsequently followed by low-molecular-weight heparin (LMWH) administration.

Following thrombolytic therapy, a further decrease in platelet count was observed, reaching a nadir of 36,000/µL. However, during the subsequent hospital course, a progressive recovery of platelet levels was noted, with values increasing to 107,000/µL at the time of discharge. This platelet recovery occurred in parallel with clinical and hemodynamic stabilization, after discontinuation of heparin and initiation of alternative anticoagulation, and was not accompanied by major bleeding complications.

**Figure 4:**
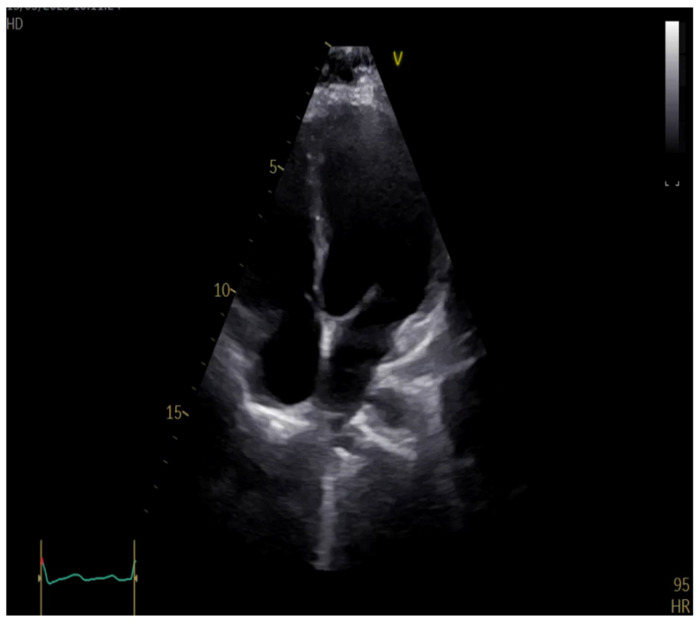
Transthoracic echocardiography at discharge showing reduction in right ventricular size compared with admission, consistent with hemodynamic improvement.

Follow-up transthoracic echocardiography after thrombolytic therapy demonstrated a reduction in right ventricular dimensions, with improvement in longitudinal systolic function, meaning a normal tricuspid annular plane systolic excursion value (TAPSE = 19 mm) and right ventricular wall motion, as shown in Figure 4. Improvement in interventricular septal kinetics, with resolution of paradoxical septal motion, was also observed. Functional tricuspid regurgitation was reduced to grade I at discharge.

The probability of heparin-induced thrombocytopenia (HIT) was evaluated using the 4Ts scoring system, which assesses thrombocytopenia, timing of platelet count decline, thrombosis, and the presence of other potential causes of thrombocytopenia [15]. In our patient, a significant reduction in platelet count was observed following heparin exposure, with a temporal pattern consistent with HIT. The occurrence of new thrombotic events, including progression to high-risk PE, further supported this assessment. Alternative causes of thrombocytopenia were considered but no alternative etiology could be clearly identified. Based on these findings, the patient was classified as having an intermediate-to-high pretest probability of HIT. Laboratory confirmation with PF4/heparin antibodies was not available at the time of decision-making and was not subsequently obtained post-discharge; therefore, HIT remained a probable clinical diagnosis.

## 4. Discussion

PE remains a leading cause of cardiovascular mortality, particularly when complicated by hemodynamic instability, as seen in the case presented above. In these high-risk clinical cases, systemic thrombolytic therapy represents a potentially life-saving treatment [16]. Because patients with thrombocytopenia are typically excluded from randomized thrombolysis trials in pulmonary embolism, the available evidence remains limited to case reports and small observational series. Nevertheless, the coexistence of thrombocytopenia poses a significant therapeutic challenge, being considered a contraindication to thrombolysis due to the associated risk of major hemorrhagic complications [17]. The evidence guiding the management of acute PE in patients with thrombocytopenia is extremely limited, consisting predominantly of isolated case reports and small case series [18,19]. As a result, clinical decision-making is largely based on individualized risk–benefit assessment rather than evidence-based recommendations [20].

The present case illustrates the successful use of systemic thrombolysis with rt-PA in a young postpartum patient with high-risk PE and moderate thrombocytopenia, in whom the imminent risk of cardiovascular collapse outweighed the potential hemorrhagic complications. By integrating this case with the available literature, this discussion aims to explore the clinical patterns and decision-making considerations associated with thrombolysis in thrombocytopenic patients with PE.

To conduct a focused and methodologically sound review starting from the reported case, the literature research encompassed articles describing high-risk PE cases where thrombolysis was the chosen therapeutic regimen in patients also reporting thrombocytopenia, irrespective of etiology. Following screening and eligibility assessment, the selected studies were included in a qualitative synthesis, summarized in Table 1.

As included in Table 1, the cases included in the qualitative synthesis involved only adults, with ages ranging from 33 years old to 77 years old and showed a clear female predominance, a finding that is consistent with the sex distribution observed in our reported case. Notably, the patient from the reported case represents one of the youngest individuals included in the review, indicating that age alone does not appear to be a risk factor in the occurrence of high-risk PE.

All patients presented with significant predisposing thrombotic risk factors, most frequently related to malignancy, with four cases reporting a history of cancer, or to recent major surgery, documented in two cases. One case was associated with thrombotic thrombocytopenic purpura, while in another patient the thrombotic etiology was attributed to COVID-19 infection. Similarly, in our case, the postpartum state following major surgery constituted a major transient prothrombotic condition, compounded by active deep venous thrombosis, underscoring the multifactorial thrombotic burden present at the time of PE.

Among the reported cases, heparin-induced thrombocytopenia (HIT) was one of the most commonly described etiologies; however, the small number of available reports precludes any definitive conclusions regarding its relative prevalence. Other causes included chemotherapy-induced thrombocytopenia, malignancy-associated platelet consumption and thrombotic thrombocytopenic purpura. In the present case, thrombocytopenia was considered compatible with suspected HIT, although platelet consumption related to the thrombotic burden cannot be completely excluded. The etiology considered in the presented patient is consistent with the high proportion of HIT reported in the existing literature.

Risk stratification in this category of patients is of critical importance for establishing the indication for thrombolytic therapy, as timely therapeutic decision-making is essential to achieve optimal clinical outcomes [29]. Formal risk stratification scores, including PESI, could not be reliably calculated retrospectively in most cases because of incomplete reporting and were available only for the index case. Therefore, PE severity was assessed using reported hemodynamic status, need for vasopressor support, and echocardiographic evidence of right ventricular dysfunction, in accordance with current guideline definitions of high-risk PE, as shown in Table 2.

Most patients included in the qualitative synthesis presented with severe clinical manifestations of PE, consistent with high-risk PE hemodynamic profile. Hemodynamic shock was reported in 75% of patients included in the review, underscoring the severe clinical presentation observed in most reported cases. Vasopressor support was also required in the cases developing hemodynamic deterioration, norepinephrine and dopamine being administered as monotherapy in 25% of the cases, whereas combined vasopressor and inotropic support was administered in two cases, 25% of the cases.

Hemodynamic instability, characterized by arterial hypotension, dyspnea, tachypnea, and tachycardia, requiring vasopressor support with norepinephrine, was also observed in our patient, aligning with the severity profiles reported in the reviewed literature. The consistent presence of hemodynamic instability across the reviewed cases suggests that thrombolytic therapy was reserved for situations of imminent life-threatening circulatory failure, despite the associated bleeding risk. Case reports and narrative reviews continue to play a crucial role in informing clinical practice in rare and high-risk presentations of pulmonary embolism, particularly in the absence of randomized data [30].

Bilateral PE was reported in 6 of the 8 patients (75%) included in the review, indicating a substantial thrombotic burden in most cases. In the remaining 2 cases (25%), the specific localization of PE could not be determined. The high prevalence of bilateral involvement among the reported patients further supports the severe clinical presentation observed in this cohort and likely contributed to the development of hemodynamic instability and right ventricular dysfunction. As described above, the patient treated in our clinic developed massive bilateral PE.

Iqbal et al. [23] reported the case where the diagnosis of PE was established based on indirect clinical and imaging findings, including the patient’s clinical deterioration with pulseless ventricular tachycardia, echocardiographic evidence of right ventricular dilatation with McConnell’s sign identified on focused assessment with sonography, and the presence of a thrombus in the inferior vena cava. Consequently, the precise localization of PE could not be determined, and it remained unclear whether the embolic involvement was unilateral or bilateral. As described by Zhu et al., the diagnosis of PE was similarly established based on indirect clinical and imaging findings, including sudden hemodynamic collapse requiring cardiopulmonary resuscitation and echocardiographic evidence suggestive of acute right ventricular overload. As comprehensive imaging confirmation was not available, the precise anatomical localization of the PE could not be established [26].

Another parameter evaluated in the present review was the presence of echocardiographic abnormalities, which play a pivotal role both in establishing the diagnosis of PE and in subsequent risk stratification. Echocardiographic findings indicative of right ventricular overload and dysfunction, such as right ventricular dilatation and reduced systolic function, assessed by parameters including tricuspid annular plane systolic excursion (TAPSE), were frequently reported. In addition, specific signs such as McConnell’s sign and abnormalities detected by tissue Doppler imaging may provide valuable diagnostic support in patients with suspected PE.

Among the analyzed cases, echocardiographic evidence of right ventricular dilatation was reported in 37,5% patients, meaning 3 of the 8 cases included in this review. Additionally, the case reported by Iqbal et al. [23] revealed severe dilatation of the right atrium and right ventricle, associated with McConnell’s sign, indicating significant acute right ventricular pressure overload. In three cases, echocardiographic findings at hospital admission were not reported. Notably, in the case described by Bethea et al. [24], initial echocardiography showed normal right ventricular function with no evidence of intracardiac thrombus; however, repeat imaging on the ninth day of hospitalization demonstrated right ventricular dilatation, suggesting subsequent hemodynamic deterioration. The same markers of severity were observed in our patient, where echocardiographic evaluation demonstrated dilatation of the right atrium and ventricle accompanied by reduced longitudinal systolic function of the right ventricle and severe functional tricuspid regurgitation.

The platelet count dynamics summarized in Table 3 highlight the substantial heterogeneity in the degree and temporal evolution of thrombocytopenia among patients with PE who underwent thrombolytic therapy. Across the reviewed cases, platelet counts at the time of thrombolysis varied widely, ranging from moderate to severe thrombocytopenia, underscoring the absence of a uniform platelet threshold guiding reperfusion decisions in real-world clinical practice.

Importantly, in several cases, systemic thrombolysis was administered at platelet levels traditionally considered contraindicating, reflecting the predominance of clinical severity over laboratory parameters when faced with imminent hemodynamic collapse. Despite low platelet counts at presentation or prior to thrombolysis, major bleeding complications were infrequently reported, suggesting that thrombocytopenia alone may not be a reliable predictor of hemorrhagic risk in this highly selected population. The most severe degree of thrombocytopenia was reported in the case published by Iqbal et al., in which thrombolytic therapy was administered at a reduced dose despite a platelet count below 10,000/µL [23]. Notably, the patient experienced significant hemodynamic improvement following thrombolysis, without major bleeding complications, with only minor mucosal bleeding from the nose and oral cavity reported.

The temporal evolution of platelet counts further suggests that thrombocytopenia was often dynamic and potentially reversible, particularly in cases attributed to HIT or consumptive mechanisms. In some reports, platelet recovery was observed following cessation of heparin exposure and initiation of alternative anticoagulation, even after thrombolytic therapy had been administered. This observation supports the notion that the etiology of thrombocytopenia, rather than the absolute platelet count alone, may be a critical determinant of bleeding risk and clinical outcome.

In our patient, thrombolysis was performed in the setting of significant thrombocytopenia (53.000/µL) with platelet values comparable to those reported in the reviewed literature. Despite this, no hemorrhagic complications were observed, and platelet counts subsequently improved during hospitalization. This clinical course parallels the favorable platelet trajectories documented in several published cases and further supports the feasibility of thrombolytic therapy in carefully selected patients with life-threatening pulmonary embolism and thrombocytopenia.

The therapeutic strategies and clinical outcomes summarized in Table 4 illustrate the considerable heterogeneity in the management of PE in patients with concomitant thrombocytopenia. Across the reviewed cases, systemic thrombolysis was the most frequently employed reperfusion strategy, predominantly using rt-PA, administered either at full or reduced doses depending on clinical severity and perceived bleeding risk. In several reports, thrombolytic therapy was initiated in the setting of profound hemodynamic compromise, including shock or cardiac arrest, underscoring its use as a rescue intervention rather than an elective treatment choice. Alirezaei et al., [21] Burns et al. [22], and Hourmouzis et al. [28] described the use of the standard rt-PA thrombolytic regimen of 100 mg administered over 2 h, the same dosage being used in the presented case. In all reported cases, thrombolytic therapy was administered as emergency treatment in the setting of hemodynamic shock, and was followed by a favorable clinical evolution, characterized by discontinuation of vasopressor and inotropic support as well as resolution of supplemental oxygen requirements.

Anticoagulation strategies following thrombolysis also varied, reflecting differences in thrombocytopenia etiology and institutional practice. In cases suspected or confirmed to involve heparin-induced thrombocytopenia, heparin was discontinued and alternative anticoagulants were initiated, whereas unfractionated or LMWH was resumed in other patients once platelet recovery was observed. These findings further highlight the importance of etiology-driven management rather than a uniform therapeutic approach. Previous studies have highlighted the feasibility of tailoring cardiovascular therapies based on clinical and echocardiographic parameters in the absence of additional invasive imaging [31].

Catheter-directed approaches were used in selected cases, reflecting attempts to mitigate bleeding risk, although evidence remains limited to individual reports. Bethea et al. [24] described a protocol involving catheter-directed administration of recombinant tissue plasminogen activator (rt-PA) via bilateral pulmonary artery catheters, delivered as a continuous infusion over 20 h. Argatroban was used both before and after thrombolysis as the anticoagulant of choice. Available evidence from the literature supports the use of argatroban as a safe and effective anticoagulant in patients with a history of heparin-induced thrombocytopenia (HIT), including during initial or repeat exposure [32,33]. A similar catheter-directed administration of recombinant tissue plasminogen activator (rt-PA) over a 20 h infusion period was also reported by Badreldin et al. [27]. In that report, systemic bivalirudin was used for anticoagulation, and thrombolytic therapy was associated with a favorable clinical outcome in a patient with massive pulmonary embolism, even though the patient experienced further complications by aspiration pneumonia.

In the case reported by Zhu et al. [26], the standard thrombolytic dosing regimen was modified to a reduced-dose protocol, consisting of a 5 mg intravenous bolus followed by 45 mg of recombinant tissue plasminogen activator (rt-PA) administered during cardiopulmonary resuscitation (CPR). Thrombolytic therapy was considered an emergency intervention in a patient with refractory cardiac arrest following 56 min of CPR. Notably, return of spontaneous circulation was achieved shortly after administration of the 5 mg rt-PA bolus, indicating a favorable reperfusion response in this extreme clinical setting.

In addition to the available case-based evidence, we reviewed current organizational, societal, and institutional guidance relevant to thrombolytic therapy. Major international guidelines, including those written by the European Society of Cardiology (ESC) and the American Heart Association (AHA), recommend systemic thrombolysis in patients with high-risk pulmonary embolism and hemodynamic instability, while emphasizing careful assessment of bleeding risk and laboratory parameters [3,34]. Within these frameworks, thrombocytopenia is generally considered a relative or absolute contraindication due to the increased risk of hemorrhagic complications; however, no specific platelet threshold is clearly defined for this clinical scenario. Importantly, many of the commonly used platelet thresholds and contraindications to thrombolytic therapy are derived from protocols developed for acute ischemic stroke. For example, a study written by Mowla et al. evaluating intravenous thrombolysis in patients with acute ischemic stroke and thrombocytopenia showed that thrombolysis may be safe even in patients with platelet counts below 100,000/mm^3^, although the available data are limited and based on a small number of cases [18]. Also, a recent systematic review, written by Ata et al., including 11 reported cases of pulmonary embolism associated with thrombocytopenia demonstrated that thrombolytic therapy may be both feasible and effective in selected patients, with a low incidence of major bleeding complications [35].

## 5. Conclusions

High-risk pulmonary embolism complicated by thrombocytopenia represents a rare but critical clinical scenario in which therapeutic decisions must balance the immediate risk of death against the potential for major bleeding. The present case, together with the qualitative synthesis of available case reports, illustrates that systemic thrombolytic therapy can be feasible and effective in carefully selected thrombocytopenic patients when life-threatening hemodynamic compromise is present.

Across the reviewed cases, thrombolysis was predominantly administered in the setting of severe clinical deterioration, including shock or cardiac arrest, often despite platelet counts traditionally considered contraindicating. Importantly, major hemorrhagic complications were uncommon, while clinical and hemodynamic improvement was frequently observed. These findings suggest that platelet count alone should not necessarily represent an absolute determinant in the decision to initiate reperfusion therapy and that clinical severity, thrombotic burden, and the underlying etiology of thrombocytopenia are critical factors guiding management. Also, reduced-dose thrombolytic strategies may represent a potential alternative in selected thrombocytopenic patients to balance efficacy and bleeding risk; however, current evidence remains limited and therapeutic decisions should be individualized.

Our case further supports this individualized approach, demonstrating favorable clinical evolution following systemic thrombolysis in a young postpartum patient with high-risk pulmonary embolism and suspected heparin-induced thrombocytopenia, without bleeding complications.

Although the available evidence is limited to case-based reports, this review highlights a consistent real-world pattern in which thrombolysis is reserved as a rescue strategy in patients with imminent circulatory collapse. This review is limited by its reliance on published case reports, which are subject to publication bias and heterogeneity in reporting. The small number of cases and absence of prospective data preclude definitive conclusions regarding safety or efficacy. In addition, incomplete reporting of prognostic variables limited retrospective risk stratification in several cases. Prospective studies are unlikely in this rare clinical context; therefore, carefully documented case reports and structured qualitative reviews remain essential to inform clinical decision-making. Multidisciplinary assessment and vigilant monitoring remain paramount when thrombolysis is considered in thrombocytopenic patients with acute pulmonary embolism.

## Figures and Tables

**Table 1:** Demographic and clinical characteristics of patients with PE and thrombocytopenia.

No	First Author/Year/Reference	Gender/Age	Documented Thrombotic Risk Factors	Suspected Cause of Thrombocytopenia
1.	Alirezaei et al., 2018 [21]	F, 65	Metastatic breast cancer	Chemotherapy
2.	Burns et al., 2009 [22]	M, 77	-Chronic myelogenous leukemia -Central venous catheter	Heparin-induced thrombocytopenia (HIT)
3.	Iqbal et al., 2023 [23]	F, 42	Lymphoma	Malignancy
4.	Bethea et al., 2017 [24]	F, 76	Cardiac surgery	HIT
5.	Soliman et al., 2022 [25]	F, 69	COVID-19 infection	HIT
6.	Zhu et al., 2015 [26]	F, 33	Thrombotic thrombocytopenic purpura	Thrombotic thrombocytopenic purpura
7.	Badreldin et al., 2018 [27]	M, 60	Pancreatic cancer	HIT
8.	Hourmouzis et al., 2015 [28]	F, 56	Laparotomy	HIT

**Table 2:** Clinical severity and hemodynamic characteristic of reported PE cases.

No	Hemodynamic Status (Shock)	Vasopressor Support	Bilateral Pulmonary Embolism	Estimated PAP (mmHg)	Echocardiography
1.	Yes	Norepinephrine	Yes	45–50	severe RV dilatation
2.	Yes	Norepinephrine Milrinone	Yes	42	severe RV dilatation
3.	Yes	Norepinephrine, dopamine, Vasopressin	Not available	55	McConnell’s sign
4.	No	No	Yes	58	Normal->RV dilatation
5.	No	No	Yes	Not available	Not available
6.	Yes	Dopamine	Not available	55	RV dilatation
7.	Yes	No	Yes	Not available	Not available
8.	Yes	No	Yes	Not available	Not available

**Table 3:** Platelet count dynamics in reported cases of PE.

No	Platelet Count at Admission (μL)	Platelet Count Pre-Thrombolysis (μL)	Lowest Platelet Count (μL)	Platelet Count at Discharge (μL)
1.	60.000	50.000	23.000	227.000
2.	798.000	73.000	24.300	766.000
3.	6.000	6.000	6.000	Not available
4.	21.000	151.000	21.000	Not available
5.	128.000	43.000	43.000	150.000
6.	23.000	23.000	23.000	Not available
7.	150.000	77.000	77.000	Not available
8.	275.000	63.000	28.000	Not available

**Table 4:** Thrombolytic treatment strategies and clinical outcomes in patients with PE and thrombocytopenia.

No	Thrombolytic Agent	Dose Regimen	Anticoagulant Before Thrombolysis	Anticoagulant After Thrombolysis	Hemodynamic Improvement	Major Bleeding
1.	rt-PA	100 mg	LMWH	UFH + LMWH	Yes	No
2.	rt-PA	100 mg	UFH	LMWH	Yes	No
3.	rt-PA	50 mg	No	No	Yes	Moderate bleeding (Nose and mouth)
4.	rt-PA	1 mg/hr (right pulmonary catheter) 2 mg/hr (left pulmonary catheter) for 3 h then 1 mg/hr for 17 h	UFH, Argotraban, Rivaroxaban	Argatroban	Yes	No
5.	rt-PA	0.9 mg/kg	Enoxaparin, UFH	UFH, Fondaparinux	Yes	No
6.	rt-PA	50 mg	No	No	Yes	No
7.	rt-PA	0.5 mg/hr (20 h)	Fondaparinux, UFH, Bivalirudin	Bivalirudin	Yes	No
8.	rt-PA	100 mg	LMWH	Argatroban	Yes	No

## Data Availability

All data are mentioned in the manuscript.

## Abbreviations

The following abbreviations are used in this manuscript:

PE	Pulmonary Embolism
RV	Right Ventricle
PESI	Pulmonary Embolism Severity Index
LMWH	Low-Molecular-Weight Heparin
HIT	Heparin Induced Thrombocytopenia
rt-PA	recombinant tissue plasminogen activator
ECG	electrocardiography
